# Tapping to Music Predicts Literacy Skills of First-Grade Children

**DOI:** 10.3389/fpsyg.2021.741540

**Published:** 2021-10-05

**Authors:** Csaba Kertész, Ferenc Honbolygó

**Affiliations:** ^1^Institute of Psychology, ELTE Eötvös Loránd University, Budapest, Hungary; ^2^Brain Imaging Centre, Research Centre for Natural Sciences, Budapest, Hungary

**Keywords:** sensorimotor synchronisation, tapping, rhythm, reading fluency, reading accuracy, phonological awareness

## Abstract

The ability to synchronise one’s movements to the sound of a regular beat has been found to be associated with children’s language and reading abilities. Sensorimotor synchronisation or tapping performance can among other factors [e.g., working memory and rapid automatized naming (RAN)] predict phonological awareness and word reading accuracy and fluency of first graders. While tapping tasks that use a simple metronome sound are more often used, applying musical stimuli has the potential advantage of being more engaging and motivating for children. In the present study, we investigated whether tapping to a metronome beat or complex musical stimuli would predict phonological awareness and reading outcomes of Hungarian 6-7-year olds (*N*=37). We also measured participants’ general cognitive abilities (RAN, non-verbal intelligence and verbal working memory). Our results show that phonological awareness, spelling and reading accuracy were associated with the musical tasks while reading fluency was predicted by the metronome trials. Our findings suggest that complex musical tasks should be considered when investigating this age group, as they were, in general, more effective in predicting literacy outcomes.

## Introduction

Moving together with the rhythm of music is a human universal (Nettl, 2000) that has been associated with the use of vocal learning and communication (Patel et al., 2009). There is also mounting evidence linking non-linguistic rhythmic abilities to literacy and reading-related cognitive skills (e.g., Moritz et al., 2013; Gordon et al., 2015; Patscheke et al., 2019). In the present study, we investigated whether a specific rhythmic ability that is synchronising one’s tapping to a steady beat could predict 6-7-year-olds’ literacy. We also compared the effectiveness of two different kinds of stimuli: the more widely used, simple metronome clicks and complex music that provides higher ecological validity.

A substantial body of research has explored the possible role of rhythmic skills in addition to well-established predictors of early literacy, such as short-term verbal memory (Peng et al., 2018), rapid automatized naming (RAN; Kirby et al., 2003) and phonological awareness (Goswami, 2018). Rhythmical skills have been associated with language and literacy investigating individuals with typical (David et al., 2007; Gordon et al., 2015; Bonacina et al., 2018; Politimou et al., 2019) and atypical language development, specifically developmental dyslexia (Flaugnacco et al., 2014; Woodruff Carr et al., 2014; Flaugnacco et al., 2015; Colling et al., 2017) and specific language impairment (Alcock et al., 2000; Corriveau and Goswami, 2009; Cumming et al., 2015).

Although there seems to be solid evidence linking rhythmic skills to reading acquisition, it is not yet clear what mediates this relationship. In their meta-analysis, Ozernov-Palchik and Patel (2018) have found that performance in especially beat-based rhythmic tasks was associated with children’s reading performance and proposed that the ability to extract regularities from an auditory stream and make predictions based on them would be the link between rhythmic abilities and phonological awareness. In the authors’ view, phonological awareness would serve as a mediator between the two domains. With similar reasoning, the Precise Auditory Timing Hypothesis (Tierney and Kraus, 2014) emphasises that both phonological skills and the ability to synchronise to a beat rely on precise neural timing and suggests that the rhythm-reading link is established through the mediation of phonology. Synchronisation on a behavioural level is widely measured using the sensorimotor synchronisation task (SMS; Repp, 2005; Repp and Su, 2013) commonly referred to as tapping to a beat.

Indeed SMS or tapping performance has been found to predict reading outcomes of Norwegian first graders (Lundetræ and Thomson, 2018) rise time perception and reading of 8-11-year-old Italian students (Flaugnacco et al., 2014), phonological processing, RAN, word reading and spelling among English speaking American 5-7-year olds (Bonacina et al., 2018) and French third graders (Lê et al., 2020). In the latter, tapping performance was not only associated with literacy scores through the mediation of phonological awareness but also showed a direct effect in the applied SEM analysis.

Building on evidence from studies with individuals with developmental dyslexia, the Temporal-Sampling Theory proposes an auditory perception framework. According to which impaired temporal processing of speech, results in imprecise phonological representations that lead to atypical reading, while the same deficit reveals itself in impaired beat processing often observed among individuals with dyslexia (Goswami, 2018). Congruent with TST, dyslexic children and adults (Wolff, 2002; Flaugnacco et al., 2014; Colling et al., 2017) and those with specific language impairment (Corriveau and Goswami, 2009; Cumming et al., 2015) have been found to be less able to synchronise to a beat.

Tapping to music, as opposed to more commonly used simple metronome clicks, has the potential benefit of engaging children more and sustaining their motivation. However, the number of studies with children using musical stimuli is scarce. In a study (Cumming et al., 2015), 9-year-old children’s tapping to music remained a significant predictor of SLI diagnosis after controlling for IQ. Although their results show that a musical task can be effective in identifying individual differences, comparison of the two stimuli was not possible due to the lack of a metronome task. Some insight may be gained from an investigation of children diagnosed with ADHD (Puyjarinet et al., 2017). When 6-12-year-old participants were asked to tap along with excerpts of classical music, their synchronisation performance deteriorated significantly compared to the metronome task. We cannot however infer that stimulus complexity has a generally negative effect on tapping performance as it could be attributable to attention deficits characteristic of ADHD. A study with contrasting results (Einarson and Trainor, 2016) compared typical developing children’s tapping to music and metronome and found that 5-7-year olds were able to synchronise their taps better when listening to music compared to metronome sound in the same tempo. A feasible explanation would be that contrary to their peers with ADHD, typical developing children can take advantage of the rhythmic complexity of music and use the richness of rhythmic cues to make temporal predictions.

In this present study, we aimed to investigate whether a musical or a metronome tapping task would be more successful in predicting children’s language and reading skills, and how stimulus complexity influences tapping performance. Based on previous studies, we hypothesised that the musical tasks would help children to synchronise to the beat resulting in more precise tapping. We could not, however, make any valid assumptions on which task would be more successful in predicting language and reading scores due to the lack of similar investigations in the literature.

## Materials and Methods

### Participants

Thirty-seven typically developing first-grade children from the Németh Imre Primary School, Budapest, Hungary took part in the current study (mean age=7.4; *SD*=0.4; 18 girls, 19 boys). All came from families that spoke Hungarian as a first language. None of them had any known neurological disorders, learning or hearing disabilities. Parents gave their written consent after being informed about the procedure. Children were also informed previously in the classroom and at the beginning of the testing sessions about the tasks and their right to withdraw their consent at any time. The study was approved by the Research Ethics Committee of Eötvös Loránd University Faculty of Education and Psychology.

### Equipment

Children were asked to tap along on an AKAI LPD8 MIDI controller, listening to music or a metronome through Audio-Technica ATH-T200 headphones, connected to a computer through a Steinberg U-22 interface. Steinberg Cubase 5 was used for playback and recording taps.

### Measurements

#### Tapping Tasks

Children were given two tapping tasks, tapping to music in three tempi (80, 120 and 150bpm) and tapping to a metronome also in the same tempi (80, 120 and 150bpm). The six trials were administered in a pseudo-random order. We used the synchronisation-continuation design in which participants were required to keep on tapping after the auditory stimulus has stopped. Children were given a short demonstration of the task which only started once they understood what they were expected to do. If a participant misunderstood the task or used an atypical strategy (e.g., tapping in antiphase or double time) they were asked to start again after a second demonstration and clarification.

##### Tapping to Music

In the musical tasks, children were instructed to listen to four quarter notes as a count-in at the beginning of each trial, then tap along with the music and continue so even after the music has ended until asked to stop. The synchronisation phase or paced tapping lasted for approximately 30s corresponding to the particular piece of music being played. In the continuation or unpaced tapping phase, participants were asked to keep tapping in the same tempo until being asked to stop (another 30s). To give participants an unambiguous sense of beat and avoid additional cues due to familiarity with the particular song, popular musical pieces were selected that are mostly unknown to Hungarian children. We also considered pop music a good choice because even though participants are not familiar with the songs themselves, the simple rhythmic and melodic motives are known to them through enculturation. Instrumental versions of three popular songs were created for the study: Dream, dream, dream (Everly Brothers), Michelle (The Beatles) and Johnny B Goode (Chuck Berry). All three were rendered from MIDI score using virtual instruments with the same instrumentation to avoid timbral differences. Vocal melody parts were removed to not give an advantage to those who were familiar with the particular song. Participants were asked if they were familiar with the particular pieces, but none of them could identify any of the songs.

##### Tapping to Metronome

Stimuli for the metronome trials were created with the same tempo and length as in the musical trials of the corresponding tempo, including the end of the synchronisation phase. For metronome sound, a woodblock sample was used from the Cubase5 library. Identical measures were calculated for the musical trials: synchronisation accuracy, synchronisation and continuation tapping consistency.

##### Synchronisation Phase

**Figure 1:**
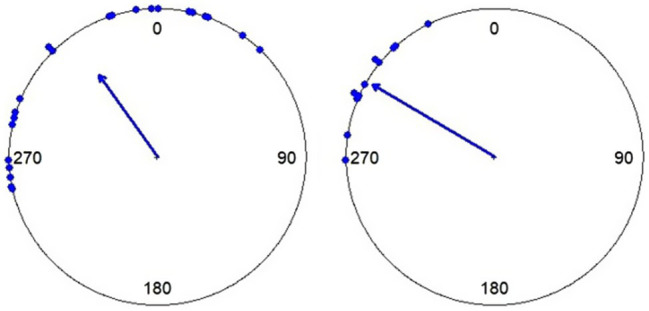
Circular plots of an individual’s tapping in two trials. Dots show single taps while the arrow represents the resultant vector. In the first case (left), taps are more consistent, shown by the arrow’s length and the mean angle signifies anticipation looking at its direction. In the second case (right), taps are less consistent while the vector’s direction shows a higher accuracy.

The first 10 taps were discarded from the analysis. Rayleigh’s tests were calculated to exclude trials in which tapping did not significantly differ from random distribution. ITIs (inter-tap intervals) for each trial were gathered for all participants to identify outliers. ITIs greater than the third quartile + three times the interquartile range (Q3+3^*^IQR) or smaller than the first quartile – three times the interquartile range (Q1–3^*^IQR) were considered outliers and were removed. After calculating the difference between each tap and the nearest target beat, two data analysis methods were applied. To measure tapping accuracy, the mean of the absolute values of differences was divided by the tempo of the music, resulting in a variable showing the deviation from the reference as a percentage, where a value of 0 means total synchrony with the stimulus. To calculate tapping consistency, circular statistical analysis was applied. Circular statistics is commonly used with cyclical or directional data (e.g., Falk et al., 2015). Individual tapping asynchronies were transformed as points on the circumference of a unit circle, representing the distance from the nearest target beat, where 0° means a perfectly timed tap. For example, in a 500ms tempo trial, a tap following the beat by 125ms would be represented by a unit vector at 90°, while the beat preceding by 125ms at −90°. The length of the resultant vector (R) averaged from the unit vectors, reflects the variability of the participant’s taps in the given trial. We used R vector length as a measure of tapping consistency, a value between 0 and 1, 0 meaning complete inconsistency and 1 meaning perfect consistency (Figure 1).

##### Continuation Phase

The continuation phase consisted of 30s of unpaced tapping immediately following the synchronisation phase. Only the first 30 taps were analysed and trials of less than 10 taps were excluded. Because of the lack of reference in this phase, circular statistics could not be applied. Tapping consistency was calculated as 1 – CV (coefficient of variance) of the ITIs, 1 meaning a hypothetical level of total consistency.

#### General Cognitive Abilities

To assess their cognitive abilities, students were administered three subtests from the fourth edition of the Wechsler Intelligence Scale for Children (Wechsler, 2003; Nagyné Réz et al., 2008): Block Design, Digit Span and Vocabulary. Age-specific standard scores were calculated and used in later analyses. The Block Design subtest was used to measure non-verbal reasoning and visuospatial abilities. Children were asked to assemble patterns presented to them on a coloured sheet, using red and white painted cubes within a given time limit. The Digit Span subtest, a measurement of verbal working memory, consisted of trials of progressively lengthening sequences of digits that children had to repeat in identical or in reverse order. Scores were calculated from the length of the longest forward and backward sequences successfully reproduced. Finally, in the Vocabulary subtest, students were asked to describe 36 words in their own way. Scores were calculated by the adequacy of their definitions using the WISC-IV manual.

#### Reading and Language Tests

Four subtests were selected from the Dyslexia Differential Diagnosis, Maastricht, Hungarian adaptation (3DM-H; Blomert and Vaessen, 2009; Tóth et al., 2014). In the Reading subtest, children were presented low- and high-frequency words and pseudowords on the computer screen. Blocks were 30s long; students were required to read as many words as possible. The length of the words and pseudowords increase gradually. Performance was assessed by fluency (how many words children were able to read) and precision (the ratio of correct answers). The Phoneme Deletion subtest required children to listen to 27 pseudowords of gradually growing complexity and repeat them excluding a certain phoneme. The percentage of correct answers was used as the measure of accuracy. In the Spelling subtest, participants were presented words through the headphones and simultaneously on the computer screen with a missing letter. They were required to complete the words using four colour coded possibilities. The percentage of correct answers was used as a measure of their performance. The Rapid Automatized Naming subtest required students to name 2 × 3 blocks of letters, numbers and pictures as quickly as possible. The performance was measured by the time used for completing a block. All measurements from the 3DM-H test are given as z-score in the analysis.

### Procedure

Children were assessed individually in two sessions, planned to take part in March of 2020. Students were administered the three tapping tasks and the three subtests from WISC-IV in a quiet, separate room. This first session (including the tapping tasks and WISC-IV) lasted approximately 40min. Participants completed reading and language tests (3DM-H) in the second session which lasted for an average of 30min. Due to the COVID-19 epidemic, the second session had to be delayed until September of 2020, the beginning of the second school year.

### Data Analysis

Data analysis was carried out using IBM SPSS Statistics, Version 23 (IBM Corp., 2015), JASP (Version 0.13.1; JASP Team, 2020) and R Studio (R Core Team, 2020). Outlier values below Q1-1,5^*^IQR or above Q3+1,5^*^IQR were identified and removed from each variable using boxplots. Paced and unpaced tapping were analysed separately. Overall tapping measurements were calculated for metronome and musical trials as the mean of the three different tempo trials resulting in six variables in total: Paced musical asynchrony, Paced metronome asynchrony, Paced musical consistency, Paced metronome consistency, Unpaced musical consistency and Unpaced metronome consistency. To compare tapping performance across musical and metronome trials, paired-sample t-tests were carried out. The assumptions of normality were tested using the Shapiro–Wilk method. The relations between tapping measurements, WISC-IV and RAN scores and reading and language outcomes were explored by building multiple linear regression models using the stepwise method. Variables were automatically entered when the value of p was equal to or lower than 0.05 and removed when above or equal to 0.10. A post-hoc power analysis was conducted using the GPower software package (Faul et al., 2007), indicating that while statistical power was more than adequate for the first two models (0.95 and 0.83), the third and fourth models showed lower levels (0.66 and 0.71) which highlight the limits of interpretation of the latter two (for parameters, see Supplementary Material).

## Results

### Differences Between Tapping to Metronome and Music

Descriptive statistics for the observed measures is shown in Table 1. To compare the tapping performance to metronome and music, paired-samples *t*-tests were conducted for variables paced tapping asynchrony, paced tapping consistency and unpaced tapping consistency. Normality assumptions were assessed using the Shapiro–Wilk test. For paced tapping consistency, there was a significant difference in the scores for musical (M=0.76, SD=0.16) and metronome (*M*=0.83, *SD*=0.09) conditions *t*(30)=−2.59, *p*=0.015; *d*=−0.47. Children tapped with higher consistency when listening to metronome sound compared to music. A significant difference was found for paced tapping asynchrony between musical (*M*=0.91, *SD*=0.06) and metronome (*M*=0.92, *SD*=0.05) conditions *t*(33)=3.02, *p*=0.005; *d*=−0.52. Participants were able to synchronise their tapping with lower asynchrony to music than to metronome. For continuation tapping consistency, there was a significant difference in performance for musical (*M*=0.09, *SD*=0.02) and metronome (*M*=0.08, *SD*=0.05) conditions *t*(32)=−3.47, *p*=0.002; *d*=−0.60. Tapping inconsistency was lower for continuation tapping following metronome than for music.

**Table 1:** Descriptive statistics for all observed measures.

Tests	*M* (*SD*)	
Spelling	−0.94	(0.63)
Phonological awareness	−0.74	(0.82)
Reading fluency	2.28	(1.00)
Reading precision	0.89	(0.09)
RAN	1.38	(0.19)
Digit span	11.06	(2.14)
Block Design	12.03	(3.48)
Vocabulary	14.68	(3.01)
Tapping consistency – music	0.76	(0.16)
Tapping consistency – metronome	0.83	(0.08)
Tapping asynchrony – music	0.12	(0.06)
Tapping asynchrony – metronome	0.15	(0.05)
Continuation consistency – music	0.91	(0.02)
Continuation consistency – metronome	0.92	(0.01)

### The Relation Between Tapping Performance, Reading and Language Scores

**Figure 2:**
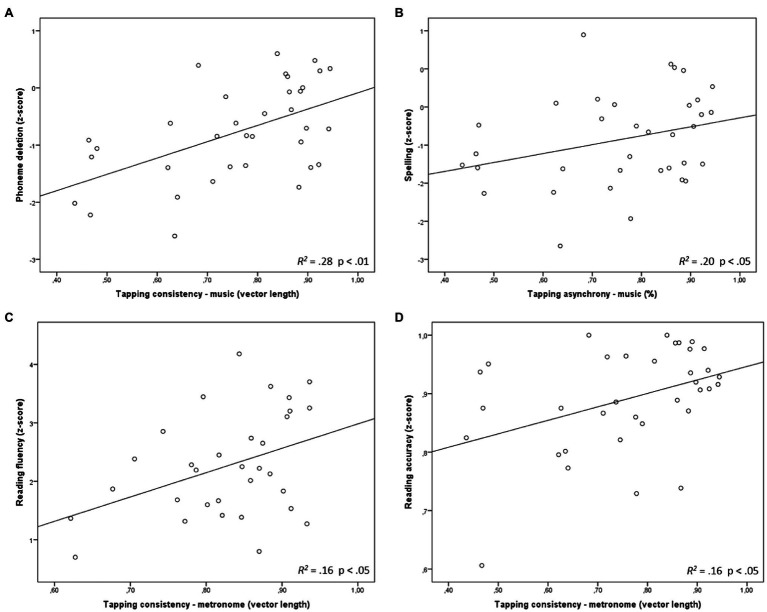
Relation between tapping consistency in musical tasks and phoneme deletion **(A)**, tapping asynchrony in musical tasks and spelling **(B)**, tapping consistency in metronome tasks and reading fluency **(C)** and tapping consistency in metronome tasks and reading accuracy **(D)**. Consistency is given as the length of the resultant vector (see Measurements), where 1 stands for perfect consistency and 0 for inconsistency. Tapping asynchrony is given as the mean asynchrony of the taps (see Measurements), where 1 represents total synchrony and 0 asynchrony.

To predict reading and language outcomes (Phoneme deletion, Reading fluency, Reading precision and Spelling), tapping variables for musical and metronome trials (Paced tapping consistency, Unpaced tapping consistency and Paced tapping accuracy), WISC-IV subtest scores (Vocabulary, Digit span and Block design) and RAN performance were entered in a series of multiple linear regression analyses using the stepwise method. The coefficients of the described linear models are summarised in Table 2 and visually represented in Figure 2. For Phoneme deletion, a significant model was found *F*(1, 28)=11.07, *p*=0.002, *R^2^*=0.28, *R^2^* Adjusted=0.26 in which Paced musical tapping consistency (*t*=3,327, *p*=0.002) was the only significant predictor (Table 2) although Block design scores almost reached the limit of significance (*p*=0.066) of inclusion in the model. The relationship between Phoneme deletion and Paced musical tapping consistency is shown in Figure 2. An analysis of standard residuals was carried out, which showed that the data contained no outliers (Std. Residual Min=−2.12, Std. Residual Max=1.91). The assumption of independent errors was met (Durbin-Watson value=2.26). The histogram of standardised residuals indicated that the distribution of errors was approximately normal, as did the P–P plot of standardised residuals, showing points that were not exactly on the line, but acceptably close. Looking at the scatterplot of standardised predicted values, we found that the data met the assumptions of homogeneity of variance and linearity. The assumption of non-zero variances was also met (Paced musical consistency, Variance=0.02; Phoneme deletion, Variance=0.67).

**Table 2:** Regression coefficients of the final models for phoneme deletion, spelling, reading fluency and reading accuracy.

Variable	Phoneme deletion				Spelling				Reading fluency				Reading accuracy			*B*	*β*	*SE*		*B*	*β*	*SE*		*B*	*β*	*SE*		*B*	*β*	*SE*
Constant	−2.88^**^		0.66	Constant	−0.34	0.25		Constant	−1.58	1.69		Constant	0.72^**^	0.08	
Tapping consistency (music)	2.81^**^	0.53	0.85	Tapping asynchrony (music)	−4.96^*^	1.87	−0.45	Tapping consistency (metronome)	4.66	2.04	0.40	Tapping consistency (music)	0.23^*^	0.10	0.40
											
*R*^2^	0.28^**^			*R*^2^	0.20^*^			*R*^2^	0.16			*R*^2^	0.16^*^		

* *p<0.05;*

** *p<0.01*

Spelling was best predicted by a model *F*(1, 28)=7.07, *p*=0.013, *R*^2^=0.20, *R*^2^_Adjusted_=0.17 with Paced musical asynchrony (*t*=−2.66, *p*=0.013) as the independent variable. The analysis of standard residuals showed that the data contained no outliers (Std. Residual Min=−2.48, Std. Residual Max=2.50) and met the assumption of independent errors (Durbin-Watson value=2.65). Examining the histogram of standardised residuals, an approximately normal distribution of errors was found. The P–P plot of standardised residuals showed an acceptable deviation from the line. The scatterplot of standardised predicted values showed that the data met the assumptions of homogeneity of variance and linearity. The assumption of non-zero variances was met (Paced musical asynchrony, Variance=0.003; Spelling, Variance=0.39).

A significant model was found *F*(1, 28)=5.23, *p*=0.030 with *R*^2^=0.16, *R*^2^_Adjusted_=0.13 to predict reading fluency with the single variable Paced metronome consistency (*t*=2.29, *p*=0.030). Analysing standard residuals we found that the data did not contain any outliers (Std. Residual Min=−1.80, Std. Residual Max=1.97). The assumption of independent errors was met (Durbin-Watson value=2.35). We found an acceptably normal distribution of errors looking at the histogram and the P–P plot of standardised residuals. The scatterplot of standardised predicted values indicated that the assumptions of homogeneity of variance and linearity were not violated. The variances of the variables in the model indicated that the assumption of non-zero variances was also met (Paced metronome consistency, Variance=0.007; Reading fluency, Variance = 0.99).

A significant regression model was found *F*(1, 28)=5.38, *p*=0.03, *R*^2^=0.16, *R*^2^_Adjusted_=0.13 in which Paced musical consistency (*t*=2.32, *p*=0.03) predicted Reading precision scores. An analysis of standard residuals showed that the data were free of outliers (Std. Residual Min=−2.72, Std. Residual Max=1.53). The assumption of independent errors was met (Durbin-Watson value=2.50). The histogram and P–P plot of standardised residuals showed approximately normally distributed errors. The scatterplot of standardised predicted values indicated that the assumptions of homogeneity of variance and linearity were not violated. The assumption of non-zero variances was also met (Paced musical consistency, Variance=0.024; Reading precision, Variance=0.008).

## Discussion

In our present study, we investigated the relationship between Hungarian first graders’ ability to tap to a beat and their literacy development, and we also studied the difference between tapping to music and tapping to a metronome. By building linear models using the stepwise method, we found that measures of tapping performance were successful in predicting literacy scores. Paced tapping consistency in musical tasks accounted for 28% of the variance in phoneme deletion and 13% in reading precision scores. Spelling was best predicted (17%) by absolute asynchrony in paced tapping in musical tasks while reading fluency by tapping consistency in paced metronome tasks (13%). Surprisingly none of the additional predictors, such as RAN, verbal working memory, vocabulary or non-verbal intelligence, were contributing significantly to the final models. We found that musical tasks were more successful in predicting language and reading scores except for reading fluency. A possible interpretation is that those children who were able to extract the beat from the musical stimuli, thus taking advantage of its higher complexity also have an advantage in extracting regularities from speech. This would be congruent with the framework of Ozernov-Palchik and Patel (2018). Our findings are also consistent with PATH framework (Tierney and Kraus, 2014) as phonological awareness was best predicted by tapping consistency. Unpaced tapping consistency, however, did not improve any of the models as we would have expected based on findings of Maróti et al. (2019). Unpaced tapping variability was altogether found to be low in our sample. A possible explanation is that children develop the capability to keep a steady beat earlier than being able to synchronise their taps to an external beat (Provasi and Bobin-Bègue, 2003; Zentner and Eerola, 2010; Provasi et al., 2014). As unpaced compared to paced tapping does not include error correction, the ability to monitor, perceive and correct one’s asynchrony might also be a key feature in predicting literacy.

Comparing children’s performance in musical and metronome tasks, we found that paced and unpaced tapping consistency was higher for metronome trials, while asynchrony was lower when tapping along with music. These findings are congruent with those of Einarson and Trainor (2016) who also found more consistent tapping but lower phase alignment for metronome trials. Similar findings were reported (Dalla Bella et al., 2017) with adults whose taps showed higher accuracy for musical trials compared to those with a metronome beat. The authors explain their findings with the well-documented phenomenon called Negative Mean Asynchrony which is the tendency for taps to precede the target beat. Although there are contrasting findings in the literature this anticipatory behaviour was found to disappear when listening to stimuli with higher rhythmical complexity (Repp, 2005; Repp and Su, 2013). Our findings suggest that musical tasks in which children have to synchronise to more complex stimuli might tap into some underlying mechanism also involved in language and reading acquisition, for example, statistical learning or executive functioning. Another important remaining question is whether indeed tapping performance predicts reading outcomes through phonological awareness or directly as in the study of Lê et al. (2020).

It should be considered that our results come from a relatively small sample which not only led to lower levels of significance in the statistical analysis but also altogether limits the generalizability of our findings. Furthermore, as mentioned before testing sessions were conducted several months apart due to the COVID-19 pandemic, making our findings somewhat less cross-sectional in nature. Furthermore, the musical stimuli of the present study represent a small proportion of styles, which might limit its generalizability. Future research might benefit from including a wider spectrum of genres. These first preliminary findings of our ongoing research raise important future issues, for example, of the role of such predictors as RAN or verbal working memory which did not account for unique variance of reading and language skills in this study.

Our current findings suggest that musical tapping tasks are not only capable of predicting reading and language outcomes of typical developing first-grade children, but also might even be more effective than tapping to metronome clicks. We suggest that future research on the relationship between sensorimotor synchronisation and literacy should include musical tasks that are of higher ecological validity and are more suitable for this age group.

## Data Availability Statement

The datasets presented in this study can be found in online repositories. The names of the repository/repositories and accession number(s) can be found at: Menedeley Data DOI: 10.17632/5b5gd7fkwc.1.

## Ethics Statement

The studies involving human participants were reviewed and approved by Research Ethics Committee (REC) Faculty of Education and Psychology of ELTE. Written informed consent to participate in this study was provided by the participants’ legal guardian/next of kin.

## Author Contributions

CK and FH conceived the presented idea. CK gathered the data and performed the statistical analyses. FH verified the analytical methods and supervised the findings of this work. All authors discussed the results and contributed to the final manuscript.

## Funding

This study was supported by the Hungarian Scientific Research Fund (project number: OTKA K 119365) and the János Bolyai Research Scholarship of the Hungarian Academy of Sciences (FH).

## Conflict of Interest

The authors declare that the research was conducted in the absence of any commercial or financial relationships that could be construed as a potential conflict of interest.

## Publisher’s Note

All claims expressed in this article are solely those of the authors and do not necessarily represent those of their affiliated organizations, or those of the publisher, the editors and the reviewers. Any product that may be evaluated in this article, or claim that may be made by its manufacturer, is not guaranteed or endorsed by the publisher.
