# Supplementary material for: Tapping to Music Predicts Literacy Skills of First-Grade Children
Source: Front Psychol. 2021 Oct 5;12:741540. doi: 10.3389/fpsyg.2021.741540 (PMC8524048; doi:10.3389/fpsyg.2021.741540)
Supplement: Supplementary file 1 [file Table_1.docx]

Supplementary Material

**Supplementary table 1.**
Parameters of post-hoc power analysis carried out on the four regression models.

|  | Sample size | Effect size (f^2^) | Power (1-β prob) |
| --- | --- | --- | --- |
| Model 1 | 35 | .39 | .95 |
| Model 2 | 36 | .25 | .83 |
| Model 3 | 32 | .19 | .66 |
| Model 4 | 35 | .19 | .71 |

Note. α = .05. The number of predictors for all models is 1.
